# Comments on Varela-Mato, V.; Cancela, J.M.; Ayan, C.; Martín, V.; Molina, A. Lifestyle and Health among Spanish University Students: Differences by Gender and Academic Discipline. *Int. J. Environ. Res. Public Health* 2012, *9*, 2728–2741

**DOI:** 10.3390/ijerph10083591

**Published:** 2013-08-13

**Authors:** Juan Jose Crespo-Salgado, Alicia Blanco-Moure

**Affiliations:** 1 Sports Medicine Specialist, University of Vigo, Pontevedra 36310, Spain; 2 Internal Medicine, Fatima Hospital of Vigo, Pontevedra 36206, Spain; E-Mail: alicia.blanco.moure@sergas.es

In regard to the article entitled “Lifestyle and health among Spanish university students: differences by gender and academic discipline” by Varela-Mato *et al.* [1] which analyzes the lifestyles of a group of students from the University of Vigo (Galicia, Spain), we would like to draw your attention to a series of disagreements with certain statements that are made therein:

1. The authors [1] transfer their results to the Spanish university with a transversal descriptive study without selection of the participants and that includes 27% of the students in one of the three campuses and does not exceed 5% of total Students at the University of Vigo, and in which nothing is known of the subjects who did not respond to the questionnaires (see “The STROBE Statement”) [2].

2. The authors use the short version of the questionnaire International Physical Activity Questionnaire (IPAQ [3]) and defined as sufficiently active to those who perform an energy expenditure greater than or equal to 1,500 Met-min/week. IPAQ itself [3] and related agencies such as the U.S. Department of Health and Human Services (USDHHS [4]), the UK National Institute of Health (NICE [5]), and the World Health Organization (WHO [6]), indicate that it is from an energy expenditure of 500 Met-min/week when defining a person as sufficiently active (equivalent to performing 150 min/week of moderate physical activity or 75 min/week vigorous physical activity).

3. With respect to the claim that the Varela-Mato *et al*. research was approved by the Ethics Committee of the University of Vigo, this cannot be because in that there is no such University Research Ethics Committee at that institution and there is only one ethics committee for the welfare of laboratory animals (SIUV [7]).

Given the circumstances, we disagree with some of the authors’ conclusions and recommend their reconsideration.
